# The value of computed tomography angiography in predicting the surgical effect and prognosis of severe traumatic brain injury

**DOI:** 10.1038/s41598-024-52385-w

**Published:** 2024-01-20

**Authors:** Junhui Chen, Wei Zhao, Xiaoming Zhu, Likun Yang, Chengjun Geng, Xu Zhang, Yuhai Wang

**Affiliations:** 1 Department of Neurosurgery, 904th Hospital of Joint Logistic Support Force of PLA, Wuxi Clinical College of Anhui Medical University, Xingyuan North Road No. 101, Liangxi District, Wuxi, 214044 Jiangsu Province China; 2 Department of Human Anatomy and Neurobiology, School of Basic Medical Science, Central South University, Changsha, Hunan Province China; 3 Department of Imaging, 904th Hospital of Joint Logistic Support Force of PLA, Wuxi Clinical College of Anhui Medical University, Wuxi, Jiangsu Province China

**Keywords:** Neuroscience, Health care, Medical research, Neurology

## Abstract

It is difficult to predict the surgical effect and outcome of severe traumatic brain injury (TBI) before surgery. This study aims to approve an evaluation method of computed tomography angiography (CTA) to predict the effect of surgery and outcome in severe TBI. Between January 2010 and January 2020, we retrospectively reviewed 358 severe TBI patients who underwent CTA at admission and reexamination. CTA data were evaluated for the presence of cerebrovascular changes, including cerebrovascular shift (CS), cerebral vasospasm (CVS), large artery occlusion (LAO), and deep venous system occlusion (DVSO). Medical records were reviewed for baseline clinical characteristics and the relationship between CTA changes and outcomes. Cerebrovascular changes were identified in 247 (69.0%) of 358 severe TBI patients; only 25 (10.12%) of them had poor outcomes, and 162 (65.6%) patients had a good recovery. Eighty-three (23.18%) patients were diagnosed with CVS, 10 (12.05%) had a good outcome, 57 (68.67%) had severe disability and 16 (19.28%) had a poor outcome. There were twenty-six (7.3%) patients who had LAO and thirty-one (8.7%) patients who had DVSO; no patients had good recovery regardless of whether they had the operation or not. Cerebrovascular injuries and changes are frequent after severe TBI and correlate closely with prognosis. CTA is an important tool in evaluating the severity, predicting the operation effect and prognosis, and guiding therapy for severe TBI. Well-designed, multicenter, randomized controlled trials are needed to evaluate the value of CTA for severe TBI in the future.

## Introduction

Traumatic brain injury (TBI) has the highest incidence of all common neurological disorders and poses a substantial public health burden, with high mortality and disability^1–8^. Maas^4^ reported that approximately half of the world's population will suffer at least one TBI in their lifetime. Data from earlier studies (1980s) in China showed that the incidence of TBI was 55.4–64.1/100,000 people per year^9^. Maas reported that the incidence was much higher in developed countries in 2017, such as America with 823.7/100,000 per year and New Zealand with 811.0/100,000 per year^4^. Certainly, with the development of the economy, the incidence of TBI in China is also increasing rapidly due to the significant increase in traffic accidents, falls, and violence^2,4,9^. Two recent China CENTER-TBI registry studies showed that more than 80% of the causes of TBI were road traffic incidents and incidental falls^9,10^.

Severe TBI is defined as a Glasgow coma scale (GCS) score ≤ 8, with higher mortality and worse outcomes. According to observational studies conducted on unselected populations, mortality rates range between 30 and 40%^4^. There are a variety of significant burdens even faced by survivors, such as rehabilitation, ventricular peritoneal shunt, and cranioplasty, which pose enormous costs to families and society. As severe TBI has different disease processes and different clinical outcomes, different approaches are required to confirm operation indications, evaluate surgery effects and prognoses, and guide management. CT is the most commonly used method to evaluate the condition of TBI. However, very little research has addressed changes in the cerebrovasculature, and most patient prognoses are evaluated just by GCS or CT examination at admission or before surgery. The relationship between cerebrovasculature after TBI and whether or not to undergo surgery and prognosis remains unclear.

CTA has high-resolution images with shorter acquisition times, and 3-D reformations have revolutionized the evaluation of trauma victims^2^. Previously, researchers addressed CTA as penetrating traumatic arterial injuries of the neck or cerebrovasculature. A recent study also showed that leakage signs in CTA had high sensitivity in the prediction of contusional hematoma expansion and were significantly associated with delayed neurological deterioration and the necessity of surgical removal^11^. However, few researchers have used CTA to evaluate general TBI without arterial injuries. The present study explored the value of CTA in predicting the surgical effect and 6-month prognosis.

## Materials and methods

### Patient population

From January 2010 to January 2020, 358 (4.1%) severe TBI patients who presented to our emergency department underwent CTA at admission and a follow-up CTA reexamination and had complete follow-up data. There were 278 men and 80 women, with an average age of 38.4 years (range 12–67 years). The time of the first CTA examination was 40 min to 6 h after trauma.

The GCS was assessed in all patients within 24 h after injury: 235 cases had GCS scores of 3–5 (most severe TBI), and 123 had GCS scores of 6–8 (severe TBI). A total of 128 patients presented bilateral pupil dilatation, and 186 patients had single pupil dilatation before the operation (Table 1).

**Table 1:** Patient population and general data.

Characteristic	Number (%)
Sex	
Male	278 (77.65%)
Female	80 (22.35%)
Age (mean)	38.4
Causes	
Motor vehicle crashes	201 (56.15%)
Falling from a height	75 (20.95%)
Violence	32 (8.94%)
Others	50 (13.96%)
GCS	
3–5	235 (61.04%)
6–8	123 (38.96%)
Pupils dilatation	
Bilateral	128 (35.75%)
Single	186 (51.96%)
None	44 (12.29%)
Intubation	109 (30.45%)
Type of hematoma^a^	
Epidural	129 (36.03%)
Subdural	171 (47.77%)
Intracerebral	183 (51.12%)
Rotterdam CT score at admission	
I–II	64 (17.88%)
III–IV	146 (40.78%)
V–VI	148 (41.34%)

Note: GCS, Glasgow coma scale.

^a^149 patients had multiple types of hematoma.

### Exclusion criteria

The exclusion criteria were the same as those in our previous study^1^. Examples include brain swelling caused by anoxia or hypotension with minor intracranial bleeding after injury, coagulation disorder or a history of aspirin intake and multiorgan malfunction, presentation without attenuated respiration and blood pressure, combination with severe injury in another bodily region, and lack of consent from family members for participation in the clinical trial. In the same clinical database and some clinical cases from another clinical trial (Controlled Decompression technique for Severe Traumatic Brain Injury, Chinese clinical trial, ChiCTR-TCC-13004002).

### Protocol approval and patient consent

This observational prospective study was conducted in the Department of Neurosurgery at PLA 904th Hospital, which is affiliated with Anhui Medical University. The protocol of the present study was approved by the Ethics Committee of PLA 904th Hospital. Verbal informed consent was obtained for all follow-up interviews, and written informed consent was obtained for outcome assessments via a postal questionnaire. All methods were performed in accordance with the relevant guidelines and regulations.

### Clinical measurements and perioperative management

All patients were managed according to the American Guidelines for the Management of Severe Traumatic Brain Injury. All patients were admitted to the neurosurgery intensive care units (NICU) after the operation or without operation, and all baseline data and medical procedures were recorded in detail by nurses and NICU physicians. All patients received CTA scans within 24 h after the operation. Important treatment decisions are made by a team of neurosurgeons and NICU physicians. These patients underwent the same treatments and routine monitoring, such as intracranial pressure (ICP) monitoring and transcranial Doppler (TCD).

### Evaluations and outcomes

The outcome was evaluated by the Glasgow Outcome Scale (GOS) score six months after TBI via a face-to-face interview according to a previous study^2^. Favorable outcome was defined as grades 4 and 5, severe disability was defined as grades 3 and 2, and poor outcome was defined as grade 1. We also compared the outcomes of subgroups of patients with cerebrovascular shift (CS), cerebral vasospasm (CVS), large artery occlusion (LAO), and deep venous system occlusion (DVSO).

**Figure 1:**
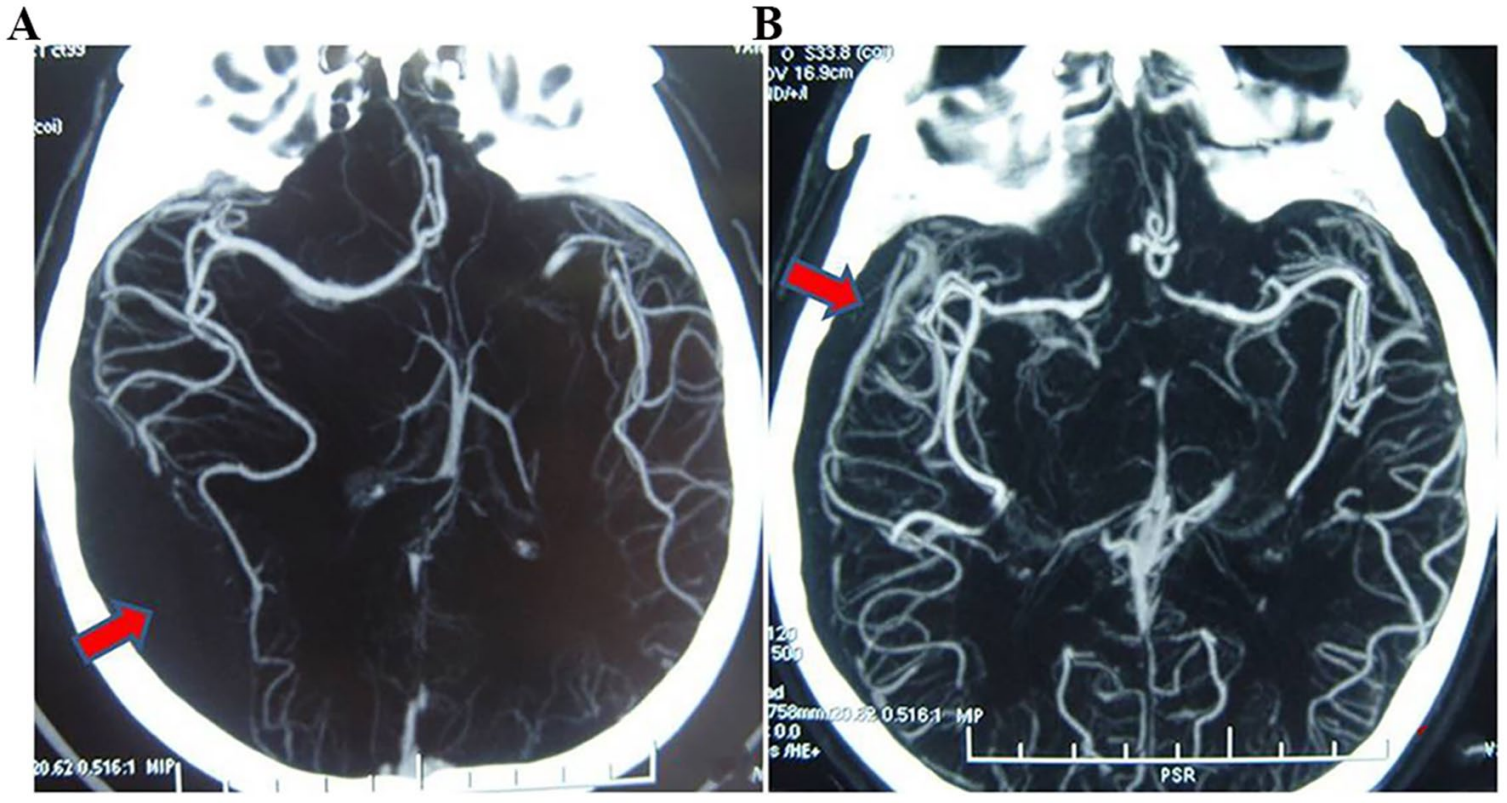
CS image of CTA. Two cases of severe TBI patients. (**A**) Acute epidural hematoma leading to CS; (**B**) acute subdural hematoma leading to CS. The red arrow indicates the hematoma.

The definition of CS in CTA was as follows: compared with contralateral cerebrovasculature, the location and vascular morphology changed (Fig. 1).

**Figure 2:**
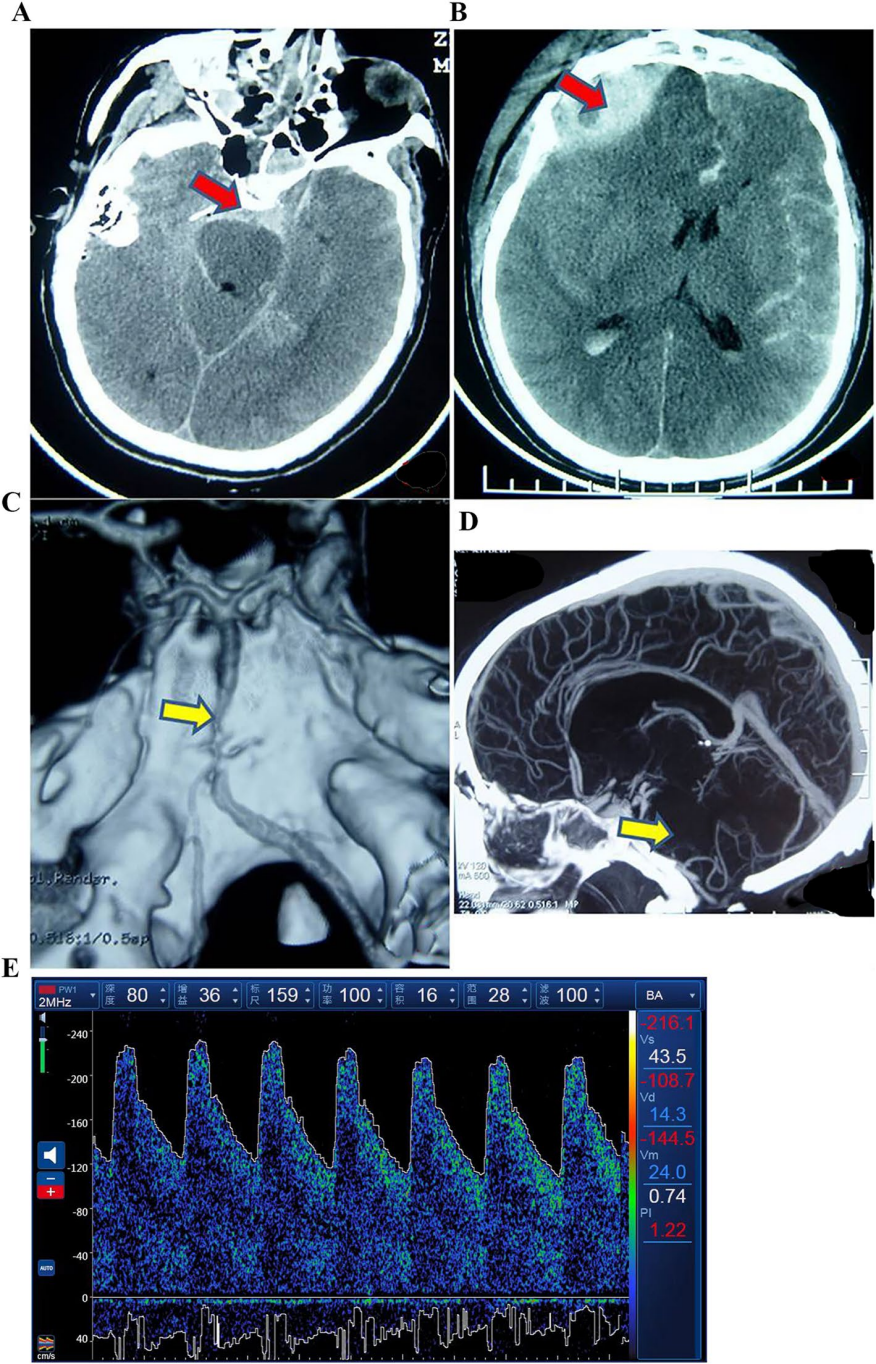
CVS image of CTA. A case of a severe TBI patient with GCS 5. (**A**) and (**B**) indicate epidural hematoma and traumatic subarachnoid hemorrhage (red arrow) by CT; (**C**) and (**D**) indicates CVS of the basilar artery and vertebral artery (yellow arrow) by CTA; (**E**) TCD showed CVS of the basilar artery.

The definition of CVS in CTA was segmental vascular stenosis^12^, and postoperative TCD and CTA examination can exclude partial intracranial arterial stenosis patients (Fig. 2).

**Figure 3:**
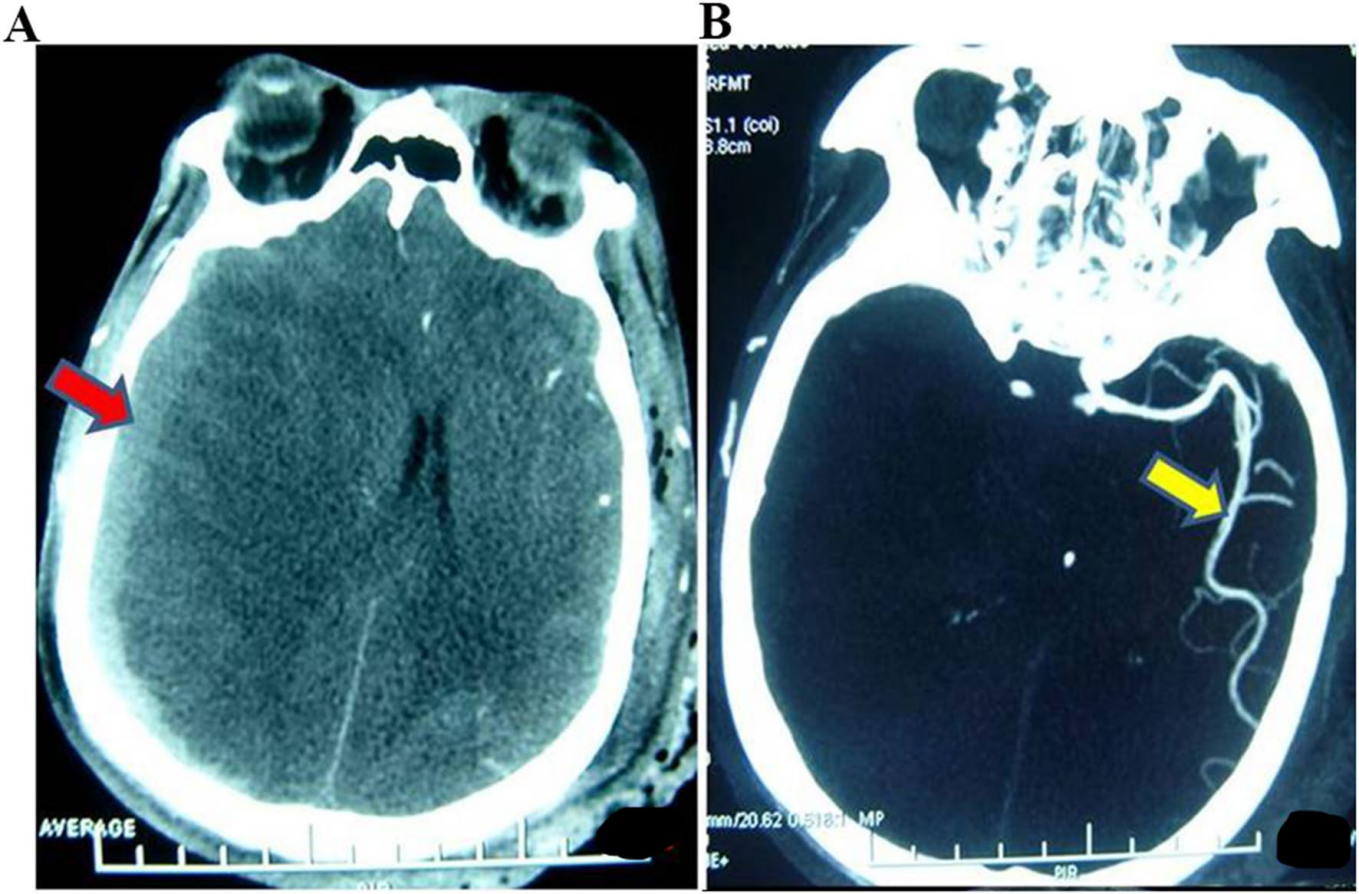
LAO of CTA. A case of a severe TBI patient with GCS 4. (**A**) CT indicates acute subdural hematoma. (**B**) CTA indicated that the right ICA and MCA were occluded.

The definition of LAO in CTA was as follows: intracranial large artery-like middle cerebral artery (MCA, including M1 and M2), internal carotid artery (ICA), arterial cerebral artery (ACA), basilar artery (BA), vertebral artery (VA), etc., no image in CTA (Fig. 3). Additionally, to exclude chronic occlusion, the patient had no history or symptoms of ischemic stroke, the occluded side matched the trauma side, and the small blood vessels could not be seen on the CTA.

**Figure 4:**
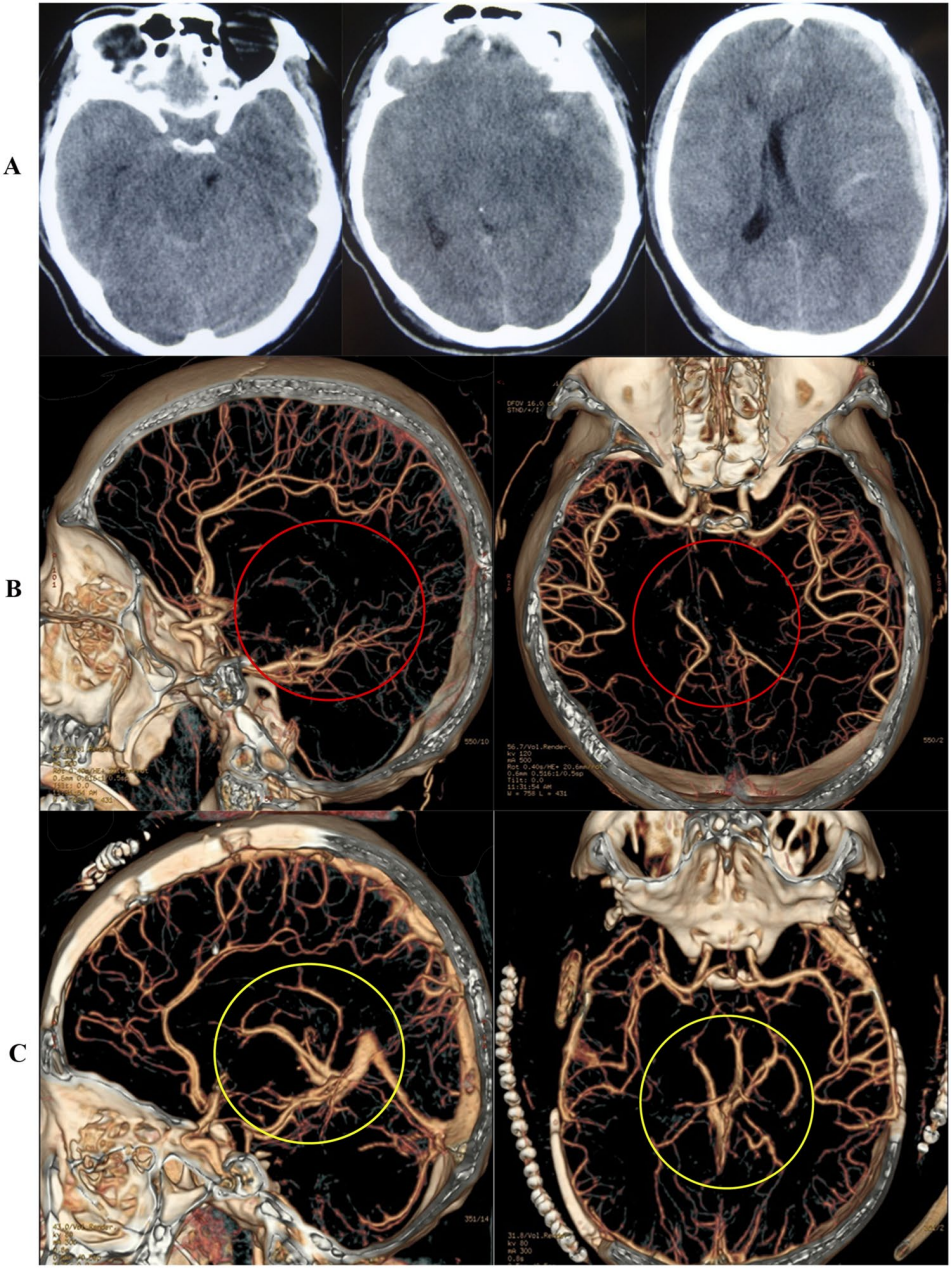
DVSO of CTA. A case of a severe TBI patient with GCS 4. (**A**) CT indicates subdural hematoma and diffuse brain swelling. (**B**) Preoperative CTA indicates intracranial deep venous system occlusion (red circle). (**C**) Postoperative CTA indicates intracranial deep venous system improvement (yellow circle).

The definition of DVSO in CTA: intracranial deep venous systems such as basilar venous, Galen venous, and cerebral internal venous disappeared (Fig. 4).

### Statistical analysis

SPSS 20.0 statistical software (SPSS, Inc., Chicago, USA) was used for the statistical analyses. An independent 2-sample t test was performed to analyze the categorical data, whereas the Pearson χ^2^ test was used for categorical variables. Ranked data were evaluated using the rank sum test. A value of *P* < 0.05 was considered statistically significant.

### Ethics statement

The study protocol was approved by the Wuxi Taihu Hospital Clinical Research Ethics Committee (2013-009). We obtained written informed consent from the family members of the patients.

## Results

### Clinical data

**Figure 5:**
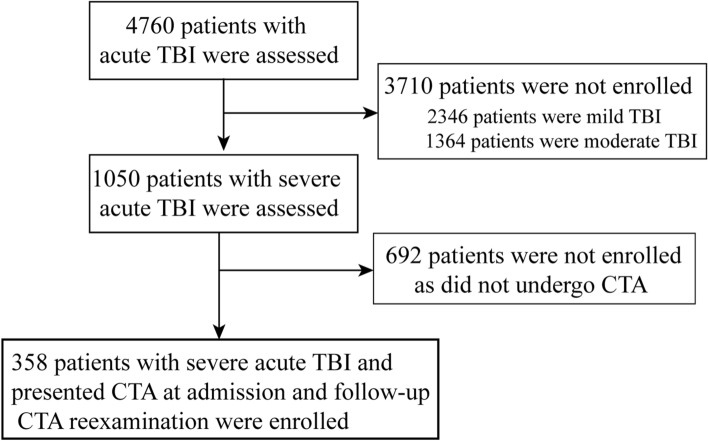
Patient’s flow chart.

From January 2010 to January 2020, a total of 4760 patients with acute TBI were admitted to our clinical institution; 3710 patients were excluded because they were diagnosed with mild or moderate TBI, and 692 patients were excluded because they did not undergo CTA examination. We retrospectively reviewed 358 severe TBI patients who presented to our emergency department and underwent CTA at admission and a follow-up CTA reexamination (Fig. 5). Among these 358 patients, 278 were men, 80 were women, and the average age was 38.4 years (ranging from 12 to 67 years). The time of the first CTA examination was 40 min to 6 h after trauma. We found that the most common causes of TBI in the present study were motor vehicle crashes (201, 56.15%), accidental falls (75, 20.95%), and violence (32, 8.94%). The GCS scores of these 358 cases were assessed within 24 h after injury as follows: 235 (61.04%) patients had GCS scores of 3–5 (most severe TBI), and 123 (38.96%) had GCS scores of 6–8 (severe TBI). There were 128 (35.75%) patients who presented bilateral pupil dilatation and 186 (51.96%) patients who had single pupil dilatation (our brain injury center included relatively severe TBI patients). (Table 1).

### CS and outcomes

CS was mostly observed after TBI by CTA. Among these 358 patients, 247 patients were diagnosed with CS by CTA, and 152/247 patients were simply CS without other vascular changes, such as CVS, LAO, or DVSO. Of these 247 patients, 39 patients presented conservative treatment, some reasons as family members refused, and the other 208 patients underwent surgery (Table 2). Under the circumstance of no difference in baseline data, the outcome of the CS group was significantly better than those without CS (*P* < 0.001), while we also found that the outcome was especially worse in the CS combined with others (one of CVS/LAO/DVSO or several) than in the simple CS group (*P* < 0.001). Compared with nonoperative CS patients (n = 39), craniotomy significantly improved the prognosis (*P* < 0.001).

**Table 2:** CS and outcome.

Group	n	Good	Disability	Poor	*P*
CS					< 0.001
Yes	247	162 (65.59%)	60 (24.29%)	25 (10.12%)	
No	111	52 (46.85%)	27 (24.32%)	32 (28.83%)	
CS & Others					< 0.001
Yes	95	29 (30.53%)	48 (50.53%)	18 (18.94%)	
No	152	133 (87.50%)	12 (7.89%)	7 (4.61%)	
CS & Operation					< 0.001
Yes	208	156 (75.00%)	38 (18.27%)	14 (6.73%)	
No	39	6 (15.38%)	22 (56.41%)	11 (28.21%)	

Note: CS, cerebrovascular shift; others: indicate one of CVS/LAO/DVSO or several.

### CVS and outcome

Among these 358 patients, 83 (23.18%) patients were diagnosed with CVS by CTA and TCD (Table 3). Of these 83 patients, 32 patients underwent conservative treatment, and the other 51 patients underwent surgery. Forty-eight CVS patients received anti-CVS drug treatment, such as fasudil, papaverine, or nimodipine, and the other 35 patients did not receive anti-CVS drug treatment. The outcome of anti-CVS drug treatment was significantly better than that without anti-CVS drug treatment (*P* = 0.003). Compared with those in the non-CVS group (275 patients), most patients in the CVS group had a significantly poorer outcome (*P* < 0.001). We also found that the outcomes of the nonoperative patients were not different from those of the operative patients (*P* = 0.586).

**Table 3:** CVS and outcome.

Group	n	Good	Disability	Poor	*P*
CVS					< 0.001
Yes	83	10 (12.05%)	57 (68.67%)	16 (19.28%)	
No	275	204 (74.18%)	30 (10.91%)	41 (14.91%)	
Anti-CVS					0.003
Yes	48	8 (16.67%)	36 (75.00%)	4 (8.33%)	
No	35	2 (5.71%)	21 (60.00%)	12 (34.29%)	
Operation					0.586
Yes	51	6 (11.76%)	34 (66.67%)	11 (21.57%)	
No	32	4 (12.50%)	23 (71.88%)	5 (15.62%)	

Anti-CVS: indicate anti-vasospasm drug and related drug treatment, include nimodipine, papaverine and fasudil.

### LAO and outcomes

There were 26 (7.26%) patients who were diagnosed with LAO by CTA; no patients had a good outcome, 3 patients had a severe disability, and the other 23 patients died. The other 332 non-LAO patients had significantly better outcomes than LAO patients (*P* < 0.001). Of these 26 patients, 20 patients underwent surgical treatment, and the other 6 patients underwent conservative treatment (Table 4). There were no significant differences between the two groups in the six-month outcome (*P* = 0.836).

**Table 4:** LAO and outcome.

Group	n	Good	Disability	Poor	*P*
LAO					< 0.001
Yes	26	0	3 (11.54%)	23 (88.46%)	
No	332	214 (64.46%)	84 (25.30%)	34 (10.24%)	
Operation					0.836
Yes	20	0	2 (10.00%)	18 (90.00%)	
No	6	0	1 (16.67%)	5 (83.33%)	

Note: LAO, large artery occlusion.

### DVSO and outcome

Of the 358 severe TBI patients, 31 (8.66%) were diagnosed with DVSO by CTA/CTV. Almost all DVSO patients had poor outcomes; 6 patients had severe disabilities, and another 25 patients died. The rates of good outcome (0% vs. 65.44%) and death (80.65% vs. 9.79%) were significantly worse than those without DVSO (*P* < 0.001). In particular, we found that the outcome of DVSO patients combined with LAO was worst, and all seven DVSO combined with LAO patients died. Nineteen (61.29%) patients underwent surgical treatment, and the other 12 patients underwent conservative treatment. However, all patients had poor outcomes with or without surgery (*P* = 0.152, Table 5).

**Table 5:** DVSO and outcome.

Group	n	Good	Disability	Poor	*P*
DVSO					< 0.001
Yes	31	0	6 (19.35%)	25 (80.65%)	
No	327	214 (65.44%)	81 (24.77%)	32 (9.79%)	
DVSO & LAO					0.341
Yes	7	0	0	7 (100.00%)	
No	24	0	6 (25.00%)	18 (75.00%)	
Operation					0.152
Yes	19	0	6 (31.58%)	13 (68.42%)	
No	12	0	0	12 (100.0%)	

Note: LAO, large artery occlusion; DVSO, deep venous system occlusion.

## Discussion

Severe TBI is currently managed in the ICU with a combined medical-surgical approach, such as physiological monitoring, autoregulation assessment, multimodal monitoring, and decompressive craniectomy. All intensive management and treatment methods aim to prevent additional brain damage and optimize conditions for brain recovery^13^. No treatments in the ICU are risk-free, and the more aggressive interventions have a substantial potential to cause harm. Cooper^14^ reported that decompressive craniectomy did not improve outcomes in the DECRA trial, and Andrews^15^ also reported that hypothermia had no outcome benefit in severe TBI patients. This may be related to the fact that the patient's primary illness was critical and did not respond well no matter what treatment option was chosen. Therefore, it is very important to choose a suitable operative patient. CTA has been intensively studied in primary and secondary intracerebral hemorrhage (ICH)^16–18^. An increasing number of studies have aimed at the application of CTA for TBI, such as traumatic cerebrovascular injury, blunt injuries of the carotid and vertebral arteries, brain tissue hypoperfusion, or hyperperfusion injury^19–21^. CTA was also used to evaluate brain blood flow and cerebral perfusion for diagnosing brain death^22–24^.

In the present study, 235 (61.04%) patients were diagnosed with extremely severe TBI (GCS 3–5), and 123 (38.96%) were diagnosed with severe TBI. Among these 358 patients, 128 (33.25%) patients experienced bilateral pupil dilatation, 186 (66.75%) patients experienced single pupil dilatation, and another 44 (12.29%) patients had good pupils. The rate of severe TBI was higher, as our hospital was the TBI center of the military. There were 247 (68.99%) patients who had CS, as most CS patients underwent craniotomy, and most of these patients had a good outcome. We found it interesting that a better outcome occurred in the CS group than in the group without CS. The reason for CS was mostly the mass effect (subdural hematoma or epidural hematoma), which occurred in many purely epidural hematomas without cerebrovascular injury and abnormal cerebral blood flow. Therefore, most patients had a good outcome if the mass effect was removed by craniotomy on time, even though they had a poor GCS score from the beginning. With the same GCS, the CS patients may be better than that of non-CS patients. In the present study, 208 patients received craniotomy, 156 patients had a good outcome, 39 patients refused craniotomy, 6 patients had a good outcome, and 11 patients died. We also found a poor outcome if CS was combined with CVS, LAO, or DVSO. Generally, there is a higher value of craniotomy and better outcomes for purely CS patients.

Secondary injury associated with severe TBI may be associated with posttraumatic vasospasm, which is a dangerous consequence of TBI^25^. Previous studies have reported that the incidence of posttraumatic vasospasm varies between 18.6% and 45.2% in the anterior circulation^26–28^. Oertel^26^ reported that 45.2% of TBI patients demonstrated at least one criterion for posttraumatic vasospasm. In the present study, 83 (23.18%) patients were confirmed to have posttraumatic vasospasm by CTA and TCD. Posttraumatic vasospasm was a significant predictor of poor outcomes ^26,29^. Lee^30^ reported that 76% of moderately severe brain injury without posttraumatic vasospasm pediatric patients had a good outcome (GCS score ≥ 4, follow-up one month after TBI), while only 40% of those with vasospasm pediatric patients had a good outcome. The rate of a good outcome was just 12.04% (10/83), which was significantly lower in patients without vasospasm. Regarding treatment strategies, some medical management strategies, such as surgery, drugs, triple-H therapy, and interventional options, have been reported to relieve posttraumatic vasospasm and improve outcomes. However, for most clinicians, craniotomy and related treatment were the primary focus, and almost no neurosurgeon initially focused on posttraumatic vasospasm. Our study found that surgery cannot improve the outcome for posttraumatic vasospasm patients, but anti-vasospasm drugs, such as nimodipine, papaverine, and fasudil, can significantly improve outcomes. Previous studies also confirmed that nimodipine can significantly reduce the incidences of death, vegetative survival, or severe disability at 6 months posttrauma compared with placebo^31^.

Traumatic large cerebrovascular injury was a serious complication and was mostly reported after severe TBI, but large artery occlusion after severe TBI was rarely reported. Kowalski^32^ reported that 6488 moderate to severe TBI survivors were enrolled, and 159 (2.5%) patients experienced an acute ischemic stroke (AIS) and found that it can predict a worse functional and cognitive outcome. Large artery occlusion was the main cause of AIS or posttraumatic cerebral infarction (PTCI). Tian^33^ reported that 11.96% of patients with moderate to severe TBI developed PTCI. Our study also showed that approximately 26 (7.3%) patients experienced LAO, only 3 patients had a disability, and the other 23 patients died regardless of the operation. Therefore, we recommend that LAO was a very important and valuable element to evaluate the value of the operation and predict the outcome. Most LAO patients have a higher ICP caused by mass effects and brain swelling^34^. Although decompressive craniectomy was a common means of rapidly reducing ICP in patients with severe TBI^2^, while no significant difference between the operative and nonoperative groups in the present study. As the data were small in the nonoperative group and with inconsistent baseline data, the result was not completely credible and needs more studies and randomized controlled trials to confirm.

DVSO has rarely been reported in previous studies, especially TBI patients. It was very important to evaluate deep venous systems, such as the basilar venous, Galen venous, and cerebral internal venous systems, before the operation by CTA/CTV. In the present study, 31 (8.7%) patients experienced DVSO, only seven patients had a disability, and the other 24 patients died. The outcome was significantly poorer than that in patients without DVSO. Almost no previous studies have reported the relationship between DVSO and the outcome after TBI. Therefore, there are no incidence or characterization data about DVSO in acute TBI patients. In our experience, the outcome was poor if any of the two deep venous occlusions by CTA were confirmed. The reasons may be as follows: deep venous occlusion indicates a higher ICP, deep venous occlusion aggravates the blockage of venous return, ICP increases, and deep venous system drainage is a very important functional area, such as the brainstem and thalamus. We can obtain deep venous system information from CTA when patients are admitted, and it is a very important imaging marker to evaluate the outcome and operation effect of severe TBI.

Our study is limited by the retrospective study’s lack of a comparison group if surgery was performed to address the issues seen on CTA for other issues. Additionally, this study was performed at a single center and lacks generalizability. We suggest that a similar, better-controlled, larger-scale, multicenter study is needed to confirm the encouraging results of our study (Chinese Clinical Trial Registry, ChiCTR2000032291).

## Conclusion

Based on the CTA image, we can obtain information on cerebrovascular shift, cerebral vasospasm, large artery occlusion, and deep venous system occlusion in severe patients. This information helped us to predict the surgical effect and prognosis of severe traumatic brain injury before surgery and to develop a better treatment strategy. However, the present study was a retrospective study, and the results were not representative. More large multicenter randomized control trials are needed to confirm these findings.

## Data Availability

All data generated or analyzed during this study are available upon reasonable request from the corresponding author.
